# Supplementary material for: Inflammatory biomarkers and perinatal depression: A systematic review
Source: PLoS One. 2024 May 31;19(5):e0280612. doi: 10.1371/journal.pone.0280612 (PMC11142563; doi:10.1371/journal.pone.0280612)
Supplement: S3 Table — (DOCX) [file pone.0280612.s004.docx]

Supplementary Table 3

| 1. **Case-control pregnancy (cross-sectional)** | | | | | | | | | | | | | | | | | | | | | | | | | | | | | | | | | | | | | | | | | | | | | | | | | | | | | | | | | | | | | | | | | |
| --- | --- | --- | --- | --- | --- | --- | --- | --- | --- | --- | --- | --- | --- | --- | --- | --- | --- | --- | --- | --- | --- | --- | --- | --- | --- | --- | --- | --- | --- | --- | --- | --- | --- | --- | --- | --- | --- | --- | --- | --- | --- | --- | --- | --- | --- | --- | --- | --- | --- | --- | --- | --- | --- | --- | --- | --- | --- | --- | --- | --- | --- | --- | --- | --- | --- |
| **Study**  **(first author/year)** | **IL-6** | **IL-10** | | **CRP** | | **IL-1β** | | **TNF-α** | | **hs_CRP** | | | | | **IL-9** | **IL-5** | **IL-12** | **IL-13** | | **IFN- γ** | | **IL-4** | **Other** | | | | | | | **IFN-α** | | **MCP-1** | | **IL-8** | | **IL-12p70** | | **IL-17A** | **IL-18** | | **IL-23** | | | **IL-33** | | | **IL-2** | | | **IL-7** | | **IL-1RA** | | **IL-17** | | | **ERVWE1** | | **Depression** | | | | | **Time points** | **Quality score** |
| Venkatesh 2019 |  |  | |  | |  | |  | |  | | | | |  |  |  |  | |  | |  |  | | | | | | |  | |  | |  | |  | |  |  | |  | | |  | | |  | | |  | |  | |  | | |  | | Perinatal depression documented by the obstetric or the primary care provider in the electronic medical record (EMR) of the study institution. | | | | | Average of three visits: 10, 18 and 26 gw | 6 |
| Cassidy-Bushrow 2012 | (+) correlation |  | |  | | (+) correlation | |  | |  | | | | |  |  |  |  | |  | |  |  | | | | | | |  | |  | |  | |  | |  |  | |  | | |  | | |  | | |  | |  | |  | | |  | | Depressive symptoms measured as CES-D total score; CES-D ≥ 16 equate with symptoms of depression; | | | | | 13.1-28.6 gw | 7 |
| Karlsson 2017 |  |  | |  | |  | |  | |  | | | | | (+) correlation | (+) correlation | (+) correlation | (+) correlation | | (+) IFN-y/IL-4 ratio | |  |  | | | | | | |  | |  | |  | |  | |  |  | |  | | |  | | |  | | |  | |  | |  | | |  | | EPDS: continuous total sum score was used for the main analyses. Additional comparisons (high/low EPDS) with the cut-point 9/10 (+ correlations only with total scores) | | | | | 24 gw | 6 |
| Christian 2009 |  |  | |  | |  | |  | |  | | | | |  |  |  |  | |  | |  |  | | | | | | |  | |  | |  | |  | |  |  | |  | | |  | | |  | | |  | |  | |  | | |  | | Depressive symptoms measured as CES-D total score (clinical cut-off 16) | | | | | M=15±7.8 gw | 7 |
| Haeri 2013 | ↑ in depressed |  | |  | |  | | ↑ in depressed | |  | | | | |  |  |  |  | |  | |  |  | | | | | | |  | |  | |  | |  | |  |  | |  | | |  | | |  | | |  | |  | |  | | |  | | DSM-V diagnosis of depression (depressed group). Documented negative EPDS for Cts | | | | | 11-14 gw (M=12.8 gw in Cts and 12.6 gw in depressed group) | 6 |
| Roomruangwong 2017 |  |  | |  | |  | |  | |  | | | | |  |  |  |  | |  | |  | ↓ IgA responses to anthranilic acid in PND | | | | | | |  | |  | |  | |  | |  |  | |  | | |  | | |  | | |  | |  | |  | | |  | | EPDS to assess the diagnosis of perinatal depression (EPDS≥11 for depressed and EPDS≤2 for non-depressed) and HAM-D | | | | | 3rd trimester (at the end of term) and 4-6 wks PP | 8 |
| Edvinsson 2017 |  |  | |  | |  | |  | |  | | | | |  |  |  |  | |  | |  | ver tabela | | | | | | |  | |  | |  | |  | |  |  | |  | | |  | | |  | | |  | |  | |  | | |  | | (1) EPDS (psychophysiology sub-study EPDS≥13, cesarean EPDS≥17). (2) MINI and MADRS performed only in participants from the psychophysiology sub-study. | | | | | (1) 17 and 32 gw; (2) 35-39 gw | 6 |
| Miller 2019 |  |  | |  | | ↑ in individuals with increased odds of PND | |  | |  | | | | |  |  |  |  | |  | |  |  | | | | | | |  | |  | |  | |  | |  |  | | ↑ in individuals with increased odds of PND | | | ↑ in individuals with increased odds of PND | | |  | | |  | |  | |  | | |  | | IDS-SR30≥18, MINI | | | | | Pre-cesarean | – |
| Chang 2018 |  |  | |  | |  | | ↑ levels in PND and (+) correlation with PND duration | |  | | | | |  |  |  |  | |  | |  |  | | | | | | |  | |  | |  | |  | |  |  | |  | | |  | | |  | | |  | |  | |  | | |  | | DSM-IV diagnosis by MINI; EPDS≥12/13 | | | | | 16-28 gw | 6 |
|  |  |  | |  | |  | |  | |  | | | | |  |  |  |  | |  | |  |  | | | | | | |  | |  | |  | |  | |  |  | |  | | |  | | |  | | |  | |  | |  | | |  | |  | | | | |  |  |
|  |  |  | |  | |  | |  | |  | | | | |  |  |  |  | |  | |  |  | | | | | | |  | |  | |  | |  | |  |  | |  | | |  | | |  | | |  | |  | |  | | |  | |  | | | | |  |  |
|  |  |  | |  | |  | |  | |  | | | | |  |  |  |  | |  | |  |  | | | | | | |  | |  | |  | |  | |  |  | |  | | |  | | |  | | |  | |  | |  | | |  | |  | | | | |  |  |
|  |  |  | |  | |  | |  | |  | | | | |  |  |  |  | |  | |  |  | | | | | | |  | |  | |  | |  | |  |  | |  | | |  | | |  | | |  | |  | |  | | |  | |  | | | | |  |  |
|  |  |  | |  | |  | |  | |  | | | | |  |  |  |  | |  | |  |  | | | | | | |  | |  | |  | |  | |  |  | |  | | |  | | |  | | |  | |  | |  | | |  | |  | | | | |  |  |
| 1. **Case-control pregnancy (cross-sectional), cont.** | | | | | | | | | | | | | | | | | | | | | | | | | | | | | | | | | | | | | | | | | | | | | | | | | | | | | | | | | | | | | | | | | |
| **Study**  **(first author/year)** | **IL-6** | **IL-10** | | **CRP** | | **IL-1β** | | **TNF-α** | | **hs_CRP** | | | | | **IL-9** | **IL-5** | **IL-12** | **IL-13** | | **IFN- γ** | | **IL-4** | **Other** | | | | | | | **IFN-α** | | **MCP-1** | | **IL-8** | | **IL-12p70** | | **IL-17A** | **IL-18** | | **IL-23** | | | **IL-33** | | | **IL-2** | | | **IL-7** | | **IL-1RA** | | **IL-17** | | | **ERVWE1** | | **Depression** | | | | | **Time points** | **Quality score** |
|  |  |  | |  | |  | |  | |  | | | | |  |  |  |  | |  | |  |  | | | | | | |  | |  | |  | |  | |  |  | |  | | |  | | |  | | |  | |  | |  | | |  | |  | | | | |  |  |
| Shelton 2015 |  |  | |  | | (-) correlations; Hierarquical linear regression models: depression contributed to the levels of plasma cytokine | | (-) correlations; Hierarquical linear regression models: depression contributed to the levels of plasma cytokine | |  | | | | |  |  |  |  | |  | |  |  | | | | | | |  | |  | |  | |  | |  |  | |  | | |  | | |  | | | (-) correlations; Hierarquical linear regression models: depression contributed to the levels of plasma cytokine | |  | |  | | |  | | POMS-D continuous total sum score (7,6% met the protocol’s screening criteria for possible clinical depression with a POMS-D score greater than 20) | | | | | 16-26 gw (M=20 gw) | 6 |
| Ruiz 2007 |  |  | |  | |  | |  | |  | | | | |  |  |  |  | |  | |  |  | | | | | | |  | |  | |  | |  | |  |  | |  | | |  | | |  | | |  | | ↑levels in group with CES-D>20 | |  | | |  | | Depressive symptoms defined as a CES-D score of >20. | | | | | 22-24 gw | 8 |
| Jallo 2021 |  |  | |  | |  | |  | |  | | | | |  |  |  |  | |  | |  |  | | | | | | |  | |  | | (+) correlation | |  | |  |  | |  | | |  | | |  | | |  | |  | |  | | |  | | CES-D continuous total sum score (CES-D ≥ 16 frequently used to indicate a positive screen for depression) | | | | | 14-17 gw | 6 |
| Serat 2020 |  |  | |  | |  | |  | |  | | | | |  |  |  |  | |  | |  |  | | | | | | |  | |  | |  | |  | |  |  | |  | | |  | | |  | | |  | |  | |  | | |  | | PMMD monitored by consultation psychiatric service. The severity of depressive symptoms was measured by the EPDS. | | | | | M= 32.9 ± 4.18 gw | 7 |
| Bianciardi 2021 |  |  | | ↑ in the PND + no-TRAUMA group compared to the HV pregnant group | |  | |  | |  | | | | |  |  |  |  | |  | |  |  | | | | | | |  | |  | |  | |  | |  |  | |  | | |  | | |  | | |  | |  | |  | | |  | | Diagnosis of PND was made by clinical interview according to the DSM-V criteria. The severity of current depressive symptoms was assessed by EPDS (EPDS≥12 for clinically relevant PND) | | | | | 22-24 gw | 6 |
| 1. **Case-control postpartum (cross-sectional)** | | | | | | | | | | | | | | | | | | | | | | | | | | | | | | | | | | | | | | | | | | | | | | | | | | | | | | | | | | | | | | | | | |
| **Study**  **(first author/year)** | **IL-6** | **TNF-α** | | **IL-8** | | **IL-1β** | **hsIL-6** | | **IL-2** | | **IFN- γ** | | | | **IL-10** | | | | | | **TGF-β2** | | **IgA** | | | **IL-1α** | | | **IL-4** | | | | | | **IL-12 p70** | | **IL-13** | | | **IL-17** | | | **IL-18** | | | **IFN-α** | | | **IL-12p40** | | **IL-5** | | | | **Outros** | | | | **Depression** | | | | | **Time points** | **Quality score** |
| Christian 2018 | (+) correlation (stimulated) | (+) correlation (stimulated) | | (+) correlation (stimulated) | |  |  | |  | |  | | | |  | | | | | |  | |  | | |  | | |  | | | | | |  | |  | | |  | | |  | | |  | | |  | |  | | | |  | | | | ES-D depressed mood subscale | | | | | 7-10 wks PP | 8 |
| Okun 2011 |  |  | |  | |  |  | |  | |  | | | |  | | | | | |  | |  | | |  | | |  | | | | | |  | |  | | |  | | |  | | |  | | |  | |  | | | |  | | | | or risk depression recurrence | | | | | 2,3,4,6,8,11,14 and 17 wks PP | 7 |
| Dong 2013 |  |  | | ↑ in PPD group | |  |  | | ↑ in PPD group | |  | | | |  | | | | | |  | |  | | |  | | |  | | | | | |  | |  | | |  | | |  | | |  | | |  | |  | | | |  | | | | patients with PPD provided by medical patterns | | | | | within the 4 weeks period of giving birth | 2 |
|  |  |  | |  | |  |  | |  | |  | | | |  | | | | | |  | |  | | |  | | |  | | | | | |  | |  | | |  | | |  | | |  | | |  | |  | | | |  | | | |  | | | | |  |  |
|  |  |  | |  | |  |  | |  | |  | | | |  | | | | | |  | |  | | |  | | |  | | | | | |  | |  | | |  | | |  | | |  | | |  | |  | | | |  | | | |  | | | | |  |  |
|  |  |  | |  | |  |  | |  | |  | | | |  | | | | | |  | |  | | |  | | |  | | | | | |  | |  | | |  | | |  | | |  | | |  | |  | | | |  | | | |  | | | | |  |  |
|  |  |  | |  | |  |  | |  | |  | | | |  | | | | | |  | |  | | |  | | |  | | | | | |  | |  | | |  | | |  | | |  | | |  | |  | | | |  | | | |  | | | | |  |  |
|  | | | | | | | | | | | | | | | | | | | | | | | | | | | | | | | | | | | | | | | | | | | | | | | | | | | | | | | | | | | | | | | | | |
| 1. **Case-control postpartum (cross-sectional), cont.** | | | | | | | | | | | | | | | | | | | | | | | | | | | | | | | | | | | | | | | | | | | | | | | | | | | | | | | | | | | | | | | | | |
| **Study**  **(first author/year)** | **IL-6** | **TNF-α** | | **IL-8** | | **IL-1β** | **hsIL-6** | | **IL-2** | | **IFN- γ** | | | | **IL-10** | | | | | | **TGF-β2** | | **IgA** | | | **IL-1α** | | | **IL-4** | | | | | | **IL-12 p70** | | **IL-13** | | | **IL-17** | | | **IL-18** | | | **IFN-α** | | | **IL-12p40** | | **IL-5** | | | | **Outros** | | | | **Depression** | | | | | **Time points** | **Quality score** |
| Groer 2006 |  |  | |  | |  |  | |  | | (-) correlation in the formula feeders | | | |  | | | | | |  | |  | | |  | | |  | | | | | |  | |  | | |  | | |  | | |  | | |  | |  | | | |  | | | | POMS-D continuous total sum score | | | | | between  4-6 wks PP (M=5.2 wks) | 8 |
| Groer 2007 |  |  | |  | |  |  | |  | | ↓ serum IFN-γ (p<.001), ↓IFN-γ/IL-10 ratio | | | |  | | | | | |  | |  | | |  | | |  | | | | | |  | |  | | |  | | |  | | |  | | |  | |  | | | |  | | | | POMS-D ≥21 (depressed mothers were categorized as those with scores in the highest decile on the POMS-D scale) | | | | | 4-6 wks PP (M=5.3 wks PP) | 6 |
| Kianbakht 2013 |  |  | |  | |  |  | |  | |  | | | |  | | | | | |  | |  | | |  | | |  | | | | | |  | |  | | |  | | |  | | |  | | |  | |  | | | | IgG, IgM and IgA and complements C3 and C4 (↑IgG levels in MDD) | | | | a psychiatrist used DSM-IV-TR to diagnose major and minor depression. | | | | | NR / immediately after parturition for inflammatory analysis | 6 |
| Achtyes 2020 | ↑ risk of PPD |  | | ↑ risk of PPD | |  |  | | ↓ risk of PPD; ↑ depressive symptoms | |  | | | | ↓serotonin and ↓quinolinic acid were associated with ↑ risk of PPD (ORserotonin = 1.43, p=.016, per nM decrease; ORquinolinic acid=4.48, p=.014, per nM decrease) and ↑ depressive symptoms (serotonin: p=.003; quinolinic acid: p=.022) ↑kynurenine/serotonin ratio was associated with an increased risk for PPD (OR = 1.35 per unit increase, p=.038) and ↓serotonin/kynurenine ratio was associated with ↑EPDS score (p=.009). | | | | | |  | |  | | |  | | |  | | | | | |  | |  | | |  | | |  | | |  | | |  | |  | | | | Tryptophan, Kynurenine; quinolinic acid; serotonin, kynurenic acid and nicotinic acid | | | | EPDS total score; clinical interview SCID-5 for diagnosis of depressive episode with peripartum onset | | | | | 8 wks PP (6-12 wks PP) | 7 |
| Kondo 2011 |  |  | |  | |  |  | |  | |  | | | |  | | | | | | ↑in mothers with depression | | ↑in mothers with depression | | |  | | |  | | | | | |  | |  | | |  | | |  | | |  | | |  | |  | | | |  | | | | EPDS ≥ 9 as having postpartum depression. | | | | | 3 mo PP | 8 |
| Fransson 2012 |  |  | |  | |  |  | |  | |  | | | |  | | | | | |  | |  | | |  | | |  | | | | | |  | |  | | |  | | |  | | |  | | |  | |  | | | |  | | | | Semi-structured interview.  Descriptions of  continuous depressed mood were considered as depressive symptoms. | | | | | Within 5 days after delivery (during labor for inflamation) | 6 |
| Groër 2005 |  |  | |  | |  |  | |  | |  | | | |  | | | | | |  | |  | | |  | | |  | | | | | |  | |  | | |  | | |  | | |  | | |  | |  | | | |  | | | | POMS-D total score (cutoff score of 25 above which triggered a referral to a mental health professional.) | | | | | 4-6 wks PP (M=4.5 ± 2.3) | 9 |
| Gruenberg 2015 |  | (-) correlations between depression and CpG-induced TNF-α | | (-) correlations between depression and RSV induced IL-8 | |  |  | |  | | (-) correlations between depression and LPS induced IFN-γ | | | | (-) correlations between depression and adaptive immune responses, namely, several DM-induced cytokines and CR-induced IL-10 | | | | | |  | |  | | |  | | | (-) correlations between depression and adaptive immune responses, namely, several DM-induced cytokines | | | | | |  | | (-) correlations between depression and adaptive immune responses, namely, several DM-induced cytokines | | |  | | |  | | |  | | |  | | (-) correlations between depression and adaptive immune responses, namely, several DM-induced cytokines | | | |  | | | | EPDS total score (EPDS≥12  indicated a need for further mental health evaluation) | | | | | 89% at 12 mo PP, 8% at 24 mo PP and 3% at 36 mo PP | 7 |
|  | | | | | | | | | | | | | | | | | | | | | | | | | | | | | | | | | | | | | | | | | | | | | | | | | | | | | | | | | | | | | | | | | |
|  | | | | | | | | | | | | | | | | | | | | | | | | | | | | | | | | | | | | | | | | | | | | | | | | | | | | | | | | | | | | | | | | | |
|  | | | | | | | | | | | | | | | | | | | | | | | | | | | | | | | | | | | | | | | | | | | | | | | | | | | | | | | | | | | | | | | | | |
|  | | | | | | | | | | | | | | | | | | | | | | | | | | | | | | | | | | | | | | | | | | | | | | | | | | | | | | | | | | | | | | | | | |
|  | | | | | | | | | | | | | | | | | | | | | | | | | | | | | | | | | | | | | | | | | | | | | | | | | | | | | | | | | | | | | | | | | |
| 1. **Longitudinal pregnancy** | | | | | | | | | | | | | | | | | | | | | | | | | | | | | | | | | | | | | | | | | | | | | | | | | | | | | | | | | | | | | | | | | |
| **Study**  **(first author/year)** | **CRP** | | | | **hsIL-6** | | | | | | | | **hsTNF-α** | | | | | **IL-6** | **TNF-α** | | | | **hsCRP** | | | | **IFN-γ** | | | | | | **IL-18** | | | | | | | **IL-8** | | **IL-10** | | **MCP-1** | | **SDF-1α** | | **MIF** | | **TRP** | **KYN** | | **Outros** | | | | | | **Depression** | | | | | **Time points** | **Quality score** |
| Azar 2013 | ↑ depressive symptoms at T1 predict ↑ CRP levels at T2; ↑ depressive symptoms at T2 predict ↑ CRP levels at T2 | | | | ↑ depressive symptoms at T2 predict ↑ IL-6 levels at T2; (+)association between increase in depressive symptoms from T1 to T2 and IL-6 levels | | | | | | | | ↑ depressive symptoms at T1 predict ↑ TNF-a levels at T2; ↑ depressive symptoms at T2 predict ↑ TNF-a at T2 | | | | |  |  | | | |  | | | |  | | | | | |  | | | | | | |  | |  | |  | |  | |  | |  |  | | (+) correlation between inflammatory markers at T1 and depressive symptoms at   T1 and T2 | | | | | | PHQ-9 continuous scores to represent symptom severity | | | | | T1 (7-10 gw), T2 (16-20 gw) | 5 |
| Blackmore 2011 |  | | | |  | | | | | | | |  | | | | |  |  | | | |  | | | |  | | | | | |  | | | | | | |  | |  | |  | |  | |  | |  |  | |  | | | | | | EPDS continuous total score, SCID (Clinical diagnoses of current depression and history of depressive episodes) | | | | | 18 and 32 gw | 6 |
| Lahti-Pulkkinen 2020 |  | | | |  | | | | | | | |  | | | | |  |  | | | | ↑ hsCRP levels in those with depressive symptoms  during pregnancy | | | |  | | | | | |  | | | | | | |  | |  | |  | |  | |  | |  |  | |  | | | | | | CES-D (continuous variable and as a binary variable indicating probable clinical depression-CES-D⩾16). | | | | | 2x wk until 38–39 gw or delivery (median 13, 19, 27 gw for inflamation) | 8 |
| Keane 2021 | ↑ CRP levels in the high-scoring group vs moderate | | | |  | | | | | | | |  | | | | |  | ↑ TNF-α in the moderate-scoring group vs the low-scoring group | | | |  | | | |  | | | | | | ↓ IL-8 levels at 20 gw vs at 15 gw for moderate and high-scoring groups; (-) correlation between IL-8 levels and EPDS scores at 20 gw | | | | | | |  | |  | |  | |  | |  | |  |  | |  | | | | | | EPDS (Lower quartile (PSS — 7.5; STAI — 23.3; EPDS — 2.5) and upper quartile scores (PSS — 17; STAI — 40; EPDS — 9.5) were used as cutoffs to define low- (<25th percentile), moderate- (25th to <75th per-centile), and high- (≥75th percentile) scoring groups for each of  the psychological evaluations. | | | | | 15 ± 1 (visit 1) and 20 ± 1 (visit 2) gws | 7 |
|  | | | | | | | | | | | | | | | | | | | | | | | | | | | | | | | | | | | | | | | | | | | | | | | | | | | | | | | | | | | | | | | | | |
| 1. **Longitudinal pregnancy to postpartum** | | | | | | | | | | | | | | | | | | | | | | | | | | | | | | | | | | | | | | | | | | | | | | | | | | | | | | | | | | | | | | | | | |
| **Study**  **(first author/year)** | **IL-6** | **CRP** | | **Trp** | | **Kyn** | | **IL-1β** | | **IL-2** | | **IL-8** | | | **IL-10** | **TNF-α** | **KYN/TRP** | **neopterin** | | **hs-CRP** | | **MCP/CCL2** | **LPS** | | **IL-6R** | | | **sgp130** | | **IL-1RA** | | **LIFR** | | **CC16** | | **IFN-γ** | | **KA** | **AA** | | **3HK** | | | **3HAA** | | | **IL-4** | | | **IL-5** | | **IL-7** | | **IL-12** | | | **IL-13** | **IL-17** | **MIP-1α** | **GM-CSF** | | **MCP-1** | **MIP-1β** | **Outros** | **Quality score** |
| Corwin 2015 |  |  | |  | |  | |  | |  | |  | | |  |  |  |  | |  | |  |  | |  | | |  | |  | |  | |  | |  | |  |  | |  | | |  | | |  | | |  | |  | |  | | |  |  |  |  | |  |  |  | 6 |
| Teshigawara 2019 |  |  | |  | |  | |  | |  | |  | | |  |  |  |  | |  | |  |  | |  | | |  | |  | |  | |  | |  | |  |  | |  | | |  | | |  | | |  | |  | |  | | |  |  |  |  | |  |  |  | 5 |
| Blackmore 2014 |  |  | |  | |  | |  | |  | |  | | |  |  |  |  | |  | |  |  | |  | | |  | |  | |  | |  | |  | |  |  | |  | | |  | | |  | | |  | |  | |  | | |  |  |  |  | |  |  |  | 5 |
| Maes 2002 |  |  | |  | |  | |  | |  | |  | | |  |  |  |  | |  | |  |  | |  | | |  | |  | |  | |  | |  | |  |  | |  | | |  | | |  | | |  | |  | |  | | |  |  |  |  | |  |  |  | 7 |
| Maes 1999 |  |  | |  | |  | |  | |  | |  | | |  |  |  |  | |  | |  |  | |  | | |  | |  | |  | |  | |  | |  |  | |  | | |  | | |  | | |  | |  | |  | | |  |  |  |  | |  |  |  | 8 |
| Maes 2001 |  |  | |  | |  | |  | |  | |  | | |  |  |  |  | |  | |  |  | |  | | |  | |  | |  | |  | |  | |  |  | |  | | |  | | |  | | |  | |  | |  | | |  |  |  |  | |  |  |  | 7 |
| Buglione-Corbett 2018 |  |  | |  | |  | |  | |  | |  | | |  |  |  |  | |  | |  |  | |  | | |  | |  | |  | |  | |  | |  |  | |  | | |  | | |  | | |  | |  | |  | | |  |  |  |  | |  |  |  | 6 |
| Paul 2019 |  |  | |  | |  | |  | |  | |  | | |  |  |  |  | |  | |  |  | |  | | |  | |  | |  | |  | |  | |  |  | |  | | |  | | |  | | |  | |  | |  | | |  |  |  |  | |  |  |  | 6 |
| Maes 2000 |  |  | |  | |  | |  | |  | |  | | |  |  |  |  | |  | |  |  | |  | | |  | |  | |  | |  | |  | |  |  | |  | | |  | | |  | | |  | |  | |  | | |  |  |  |  | |  |  |  | 6 |
| Zhou 2018 |  |  | |  | |  | |  | |  | |  | | |  |  |  |  | |  | |  |  | |  | | |  | |  | |  | |  | |  | |  |  | |  | | |  | | |  | | |  | |  | |  | | |  |  |  |  | |  |  |  | 8 |
| Bränn 2017 |  |  | |  | |  | |  | |  | |  | | |  |  |  |  | |  | |  |  | |  | | |  | |  | |  | |  | |  | |  |  | |  | | |  | | |  | | |  | |  | |  | | |  |  |  |  | |  |  | (Note1) | 6 |
| Osborne 2019 |  |  | |  | |  | |  | |  | |  | | |  |  |  |  | |  | |  |  | |  | | |  | |  | |  | |  | |  | |  |  | |  | | |  | | |  | | |  | |  | |  | | |  |  |  |  | |  |  | 23 cytokines | 7 |
| Roomruangwong 2017 |  |  | |  | |  | |  | |  | |  | | |  |  |  |  | |  | |  |  | |  | | |  | |  | |  | |  | |  | |  |  | |  | | |  | | |  | | |  | |  | |  | | |  |  |  |  | |  |  |  | 6 |
| Scrandis 2008 |  |  | |  | |  | |  | |  | |  | | |  |  |  |  | |  | |  |  | |  | | |  | |  | |  | |  | |  | |  |  | |  | | |  | | |  | | |  | |  | |  | | |  |  |  |  | |  |  |  | 6 |
| Cheng 2014 |  |  | |  | |  | |  | |  | |  | | |  |  |  |  | |  | |  |  | |  | | |  | |  | |  | |  | |  | |  |  | |  | | |  | | |  | | |  | |  | |  | | |  |  |  |  | |  |  |  | 4 |
| Krause 2014 |  |  | |  | |  | |  | |  | |  | | |  |  |  |  | |  | |  |  | |  | | |  | |  | |  | |  | |  | |  |  | |  | | |  | | |  | | |  | |  | |  | | |  |  |  |  | |  |  |  | 7 |
| Scrandis 2008, 2010 |  |  | |  | |  | |  | |  | |  | | |  |  |  |  | |  | |  |  | |  | | |  | |  | |  | |  | |  | |  |  | |  | | |  | | |  | | |  | |  | |  | | |  |  |  |  | |  |  |  | 6 |
| Simpson 2016 |  |  | |  | |  | |  | |  | |  | | |  |  |  |  | |  | |  |  | |  | | |  | |  | |  | |  | |  | |  |  | |  | | |  | | |  | | |  | |  | |  | | |  |  |  |  | |  |  |  | 7 |
| Skalkidou 2009 |  |  | |  | |  | |  | |  | |  | | |  |  |  |  | |  | |  |  | |  | | |  | |  | |  | |  | |  | |  |  | |  | | |  | | |  | | |  | |  | |  | | |  |  |  |  | |  |  |  | 4 |
| Sha 2022 |  |  | |  | |  | |  | |  | |  | | |  |  |  |  | |  | |  |  | |  | | |  | |  | |  | |  | |  | |  |  | |  | | |  | | |  | | |  | |  | |  | | |  |  |  |  | |  |  |  | 7 |
| Nazzari 2020a |  |  | |  | |  | |  | |  | |  | | |  |  |  |  | |  | |  |  | |  | | |  | |  | |  | |  | |  | |  |  | |  | | |  | | |  | | |  | |  | |  | | |  |  |  |  | |  |  |  | 7 |
| Nazzari 2020b |  |  | |  | |  | |  | |  | |  | | |  |  |  |  | |  | |  |  | |  | | |  | |  | |  | |  | |  | |  |  | |  | | |  | | |  | | |  | |  | |  | | |  |  |  |  | |  |  |  | 7 |
| 1. **Longitudinal postpartum** | | | | | | | | | | | | | | | | | | | | | | | | | | | | | | | | | | | | | | | | | | | | | | | | | | | | | | | | | | | | | | | | | |
| **Study**  **(first author/year)** | **CRP** | | **Outro** | | | | | | | | | | | **hs-CRP** | | | | | | | | | | **IL-6** | | | **IL-1β** | | | | **TNF-α** | | | | | | | | | | | | | | **Depression** | | | | | | | | | | | **Time points** | | | | | **Time points for inflammation** | | | | **Quality score** |
| Boufidou 2009 |  | |  | | | | | | | | | | | (+) correlations between CSF TNF-α and IL-6 as well as serum TNF-α levels with EPDS scores in early puerperium.  1st wk PP: ↑CSF IL-6 (p=.039*); ↑Serum TNF-α (p=.055*) and ↑CSF TNF-α (p=.009*) were predictors of ↑EPDS.  6th wk PP: ↑CSF IL-6 (p=.012*) and ↑CSF TNF-α (p=.072*) were predictors of ↑EPDS. *statistical significance (p<.100) | | | | | | | | | |  | | |  | | | | (+) correlations between CSF TNF-α and IL-6 as well as serum TNF-α levels with EPDS scores in early puerperium.  1st wk PP: ↑CSF IL-6 (p=.039*); ↑Serum TNF-α (p=.055*) and ↑CSF TNF-α (p=.009*) were predictors of ↑EPDS.  6th wk PP: ↑CSF IL-6 (p=.012*) and ↑CSF TNF-α (p=.072*) were predictors of ↑EPDS. *statistical significance (p<.100) | | | | | | | | | | | | | | EPDS≥11 | | | | | | | | | | | 1st wk (day 4) and 6th wks PP | | | | | (1) early in labor; (2) right before epidural analgesia was infused | | | | 6 |
| Corwin 2008 |  | |  | | | | | | | | | | |  | | | | | | | | | |  | | | women with depressive symptoms on Day 28 had ↑ IL-1β levels on day 14 PP | | | |  | | | | | | | | | | | | | | CES-D≥11 (36% were identified as demonstrating symptoms of depression on 28 day PP) | | | | | | | | | | | 28 days PP | | | | | 0 (within 24 hr of giving birth), 7, 14 and 28 days PP | | | | 6 |
| Liu 2016 |  | |  | | | | | | | | | | |  | | | | | | | | | |  | | |  | | | |  | | | | | | | | | | | | | | EPDS≥12 | | | | | | | | | | | within 6-mo after delivery | | | | | Within 48 hours of delivery | | | | 8 |
| Bränn 2020 |  | | 70 of 92 inflammatory markers (21 excluded for not having normalized protein expression for >50% of the participants and 1 for technical problems) | | | | | | | | | | |  | | | | | | | | | |  | | |  | | | |  | | | | | | | | | | | | | | EPDS ≥ 12 and/or MINI interview (taking antidepressants was also used to identify cases) | | | | | | | | | | | 6 or 8 wks PP | | | | | Days from delivery (M±SD): Cts=69.5±9.7; PPD-symptoms: 67.8±11.1 | | | | 6 |
| Miller 2019 |  | |  | | | | | | | | | | |  | | | | | | | | | |  | | |  | | | |  | | | | | | | | | | | | | | SIGH-ADS29 | | | | | | | | | | | baseline (study entry), study exit (4-8 wks post-study entry) | | | | | baseline (study entry), study exit (4-8 wks post-study entry) | | | | 6 |
| *Note1: 74 of 92 inflammatory markers (16 excluded for being below LOD for >50% of the samples and 2 for technical problems); Note 2: Red: negative association- Gree: positive association- Grey: no association found* | | | | | | | | | | | | | | | | | | | | | | | | | | | | | | | | | | | | | | | | | | | | | | | | | | | | | | | | | | | | | | | | | |
