# Supplementary material for: Inflammatory biomarkers and perinatal depression: A systematic review
Source: PLoS One. 2024 May 31;19(5):e0280612. doi: 10.1371/journal.pone.0280612 (PMC11142563; doi:10.1371/journal.pone.0280612)
Supplement: Attachment — Submitted filename: Response to Reviewers.docx [file pone.0280612.s005.docx]

Manuscript ID: PONE-D-23-00135

Title: Inflammatory biomarkers and perinatal depression: a systematic review

Journal: PLOS One

Braga, August 07, 2023

Dear Editor, Liliana G Ciobanu,

We are delighted to have been given the opportunity to revise our work. Positive comments from the reviewers, such as Reviewer 1 stating that “This review is a well-argued paper that addresses an important and very relevant issue to clinical practice. Also, the methodology is very rigorous” further support us in considering this study, undertaken by experts within the EU COST action RiseUp PPD, as an important contribution in the field. Despite slightly similar works published recently, we approach the subject from angles that have not been considered yet. We would also like to deeply acknowledge the time and effort provided to this review that has significantly improved the quality of our manuscript.

Please find enclosed the revised version of our article “Inflammatory biomarkers and perinatal depression: a systematic review”, by Anabela Silva-Fernandes, Ana Conde, Margarida Marques, Rafael A. Caparros-Gonzalez, Emma Fransson, Ana Raquel Mesquita, Bárbara Figueiredo, Alkistis Skalkidou, for your consideration for publication to PLOS One.

We have strived to integrate all the feedback and suggestions provided and addressed each of the comments provided. All alterations are marked as tracked changes in the attached revised manuscript. Please find below a detailed description on how we address the Editor and Reviewers’ comments. We hope that you will find the improved manuscript suitable for publication in PLOS One.

As requested, we state that “The funders had no role in study design, data collection and analysis, decision to publish, or preparation of the manuscript”.

Yours sincerely,

Alkistis Skalkidou, MD, PhD

Professor of Obstetrics and Gynecology, Uppsala University, Sweden

Corresponding Author

**Comments and responses to the Editor and reviewers:**

**Editor and Journal requirements:**

When submitting your revision, we need you to address these additional requirements.

1. Please ensure that your manuscript meets PLOS ONE's style requirements, including those for file naming. The PLOS ONE style templates can be found at

https://journals.plos.org/plosone/s/file?id=wjVg/PLOSOne_formatting_sample_main_body.pdf and https://journals.plos.org/plosone/s/file?id=ba62/PLOSOne_formatting_sample_title_authors_affiliations.pdf

**R: We thank you for this recommendation. We revised the entire manuscript to be in line with PLOS style format.**

2. Thank you for stating the following financial disclosure:

"AS-F was supported by the Portuguese Foundation for Science and Technology and the Portuguese Ministry of Science, Technology and Higher Education, through the national funds, within the scope of the Transitory Disposition of the Decree No. 57/2016, of 29th of August, amended by Law No. 57/2017 of 19 July and previously through the fellowship grant SFRH/BPD/107732/2015. This paper is part of the COST Action Riseup-PPD CA18138 and was supported by COST under COST Action Riseup-PPD CA18138 (Virtual Mobility Grant)."

Please state what role the funders took in the study. If the funders had no role, please state: "The funders had no role in study design, data collection and analysis, decision to publish, or preparation of the manuscript."

If this statement is not correct you must amend it as needed.

Please include this amended Role of Funder statement in your cover letter; we will change the online submission form on your behalf.

**R: Dear Editor, in response to journal requirements we inform that funders had no role in the study and we have now included the statement as well as some more information in the appropriate section, the text now reading: AS-F was supported by the Portuguese Foundation for Science and Technology and the Portuguese Ministry of Science, Technology and Higher Education, through the national funds, within the scope of the Transitory Disposition of the Decree No. 57/2016, of 29th of August, amended by Law No. 57/2017 of 19 July and previously through the fellowship grant SFRH/BPD/107732/2015. AS has received funding from the Swedish Research Council, (Dnr. 2020-01965) and the Swedish Brain Foundation (FO2021-0161, Dnr. UU-PROJ 2021/332).**

**This paper is part of the COST Action Riseup-PPD CA18138 and was supported by COST under COST Action Riseup-PPD CA18138 (Virtual Mobility Grant). The funders had no role in study design, data collection and analysis, decision to publish, or preparation of the manuscript.**

**Information on the COST Action (European Cooperation in Science and Technology) are also provided in the revised manuscript, at page 26-27. Due to institutional matters, the name of the research center of the Ana Conde have changed, so we kindly ask that the institutional affiliation of this author can be changed, also corrected in the revised manuscript (title page, line 17).**

Reviewer #1: I appreciate having read this article and being able to comment. This review is a well-argued paper that addresses an important and very relevant issue to clinical practice. Also, the methodology is very rigorous.

However, some minor amendments are necessary:

1. In the introduction, include more precise information on the relationship between depression and inflammatory biomarkers

**R: We thank you for this recommendation. We have updated the Introduction with more information about the relationship between depression and inflammatory biomarkers, that consists of a proxy of the immune response (please see pages 4-5 in the revised manuscript, line 97-112).**

2. Please provide information about Newcastle-Ottawa Scale. Why this scale has been used?

**R: We thank you for this question. The Newcastle Ottawa scale (NOS) assesses the quality of nonrandomized studies, including case-control and cohort studies, which fits with the study designs of the studies selected for this systematic review. In this manner, we have used three different scale forms accordingly the study design: case-control studies and cohort studies (Wells, G. A., Shea, B., O’Connell, D., Peterson, J., Welch, V., Losos, M., & Tugwell, P. (2000) as well as an adapted form for cross-sectional studies (Herzog, R., Álvarez-Pasquin, M.J., Díaz, C. et al. Are healthcare workers’ intentions to vaccinate related to their knowledge, beliefs and attitudes? a systematic review. BMC Public Health 13, 154 (2013).** [**https://doi.org/10.1186/1471-2458-13-154**](https://doi.org/10.1186/1471-2458-13-154)**). This scale is widely used in this kind of work. More detailed information about this Scale was included on the page 11, line 250-258. If the Reviewer wishes us to expand this information, we would gladly do so.**

Reviewer #2: PONE-D-23-00135

Thanks for giving me the opportunity to read this paper, which systematically reviews available literature around an important topic concerning the association between inflammation and depression during the perinatal period. The authors conducted their search on three electronic databases and retrieve 3527 references. At the final stage 83 studies were included in the review and 56 were directly assessed as their primary aim was related to investigating the association between perinatal depression and inflammation. Results highlight several associations between a number of inflammatory markers and depressive symptoms either antenatally or postnatally, although high methodological heterogeneity limits comparison among studies. The topic and issues addressed by the current paper are interesting and important. One main criticisms concern the fact that a number of systematic reviews already exist on this topic (some examples below). I think it is essential that the authors explicitly state in what ways their work extends those reviews and adds to the available literature. Also, a more systematic / critical approach is needed in the results and discussion section to enable the reader to properly judge the weight of the available evidence. For example, the authors performed the quality assessment of the studies but this is not mentioned later when presenting the results.

Please find my major concerns better outlined below

1. Introduction: on page 5 it is stated that “This intensification of pro-inflammatory activity in late pregnancy co-occurs with an increase in depressive symptoms during this period, compared to lower levels in the second trimester”. I found this part confusing - an increase in levels of pro-inflammatory markers is to some extent physiological and normative across the third trimester of pregnancy, whereas greater than typical elevations have been associated with dep symptoms. I would suggest to have two different paragraph 1) concerning normative physiological changes that occur across the perinatal period in the immune system functioning 2) outline possible associations with mood symptoms.

**R: We thank the reviewer for pointing out the need for clarification. We have re-written this section (please see pages 6-7, line 141-155), with the aim to highlight that although this process is a universal and physiological process during pregnancy, there could be a) individual differences in the sensitivity to this fluctuation, where some individuals react with mood symptoms with changes in inflammatory parameters and b) the level of inflammatory reactions could be further elevated in individuals with, for example, pregnancy depressive symptoms, who have been exposed to childhood traumatic events, or they might be differing by personality type.**

2. Several reviews have been already conducted on this topic – some examples are:

• McCormack, C., Abuaish, S., & Monk, C. (2023). Is There an Inflammatory Profile of Perinatal Depression?. Current Psychiatry Reports, 1-16.

• Sawyer KM. The role of inflammation in the pathogenesis of perinatal depression and offspring outcomes. Brain, Behavior, & Immunity-Health. 2021;18:100390.

• Lambert M, Gressier F. Inflammatory biomarkers and postpartum depression: a systematic review of literature. Canadian Journal of psychiatry 2019;64(7):471-81.

• Leff-Gelman, P., Mancilla-Herrera, I., Flores-Ramos, M., Cruz-Fuentes, C., Reyes-Grajeda, J. P., García-Cuétara, M. D. P., ... & Pulido-Ascencio, D. E. (2016). The immune system and the role of inflammation in perinatal depression. Neuroscience bulletin, 32, 398-420.

• Osborne, L. M., & Monk, C. (2013). Perinatal depression—the fourth inflammatory morbidity of pregnancy?: theory and literature review. Psychoneuroendocrinology, 38(10), 1929-1952.

In this sense it needs to be clearly stated what the current review adds to the prior available work. Also, considering that a number of reviews has been already performed and there is a consistent number of potentially available studies to include why not thinking about performing a meta-analysis?

**R: Thank you for this very relevant question. Although several reviews have been already conducted on this topic, not all have systematically analyzed the association between inflammation and depression during pregnancy (cross-sectionally), during the postpartum (cross-sectionally) and from pregnancy to the postpartum period. Our systematic review intended to present the diversity of studies aiming to analyze associations between perinatal depression and inflammatory biomarkers with special focus both on the study design (cross-sectional, case-control and longitudinal studies) as well as, and even more importantly, on the exact period of biomarker and depression assessment (pregnancy and/or postpartum). We even included a separate note on prospective longitudinal studies, aiming to explore the possibility of a predictive role of either depression on later inflammation profiles or conversely, of inflammatory molecules on the later development of depression, which often not specifically and systematically addressed. At the end of the introduction (pages 7-8) we now specifically describe the novelty of this systematic review in relation to the other reviews named by the Reviewer (some of them already addressed in the manuscript) and updated with the references of the newly published systematic review published by McCormack, C et al. (2023) and the narrative reviews of Leff-Gelman et al. (2016) and Osborne & Monk (2013).**

**As stated on page 26, the diversity of assessment methods for the different inflammatory markers, for the clinical or subclinical depressive symptoms in different time points (pregnancy, delivery and postpartum) makes a quantitative synthesis of the results in the context of a meta-analysis nearly impossible. Nevertheless, some considerations about the usefulness of a meta-analysis, namely focused on specific more prominent biomarkers are now included in the discussion section, page 26, line 624-634.**

3. On page 7 it is stated: “(2) if one of the variables could be used as a predictor for the other”. Please explain.

**R: Thank you for this suggestion. We have re-formulated the study aims at the end of the introduction (please see page 8, line 190-194). The text now reads “(2) focusing on longitudinal studies, to explore the possibility of a predictive role of either depression on later inflammation profiles or conversely, of inflammatory molecules on the later development of depression.”**

4. Studies selection: please specify the time range considered for the “postpartum period” on page 8.

**R: Thank you for this question. We now added in the section “Studies Selection”, page 9, line 219, the time range considered for the postpartum period. The text now reads “(up to one year after delivery)”.**

5. Data extraction (on page 9): were information concerning possible medication/pharmacological treatment also available, extracted and assessed?

**R: Thank you for this question. Medications/pharmacological treatment was not included as variable of interest in our data extraction. We agree that such data is of great consequence, so this missing information is now included in the limitations section (line 619, page 25).**

6. Please describe how the quality assessment was performed, which is the score range and how it is attributed to a study.

**R: Thank you for this suggestion. We add this information in the methods (page 11, line 250-258) and results (page 12-13, line 297-309).**

7. Please state sample size range for the studies included

**R: Thank you for this suggestion. We add on Results section page 12, line 289-292, the sample size range for cross-sectional and case-control studies, as well as for longitudinal studies. The text now reads “according to the period involved (pregnancy or postpartum) (for details see Table 1) involving a sample size range between 10-469. Secondly, longitudinal studies are presented, with a sample size ranging from 26 to 467, with a note on whether a study included predictive analyses (for details see Table 2).)”**

8. The results section needs to be substantially revised in an effort to summarize the available literature. Please be more specific and systematic in the description of the studies to allow the reader to judge the relatively weight of the evidence. For example: Which is the sample size range? How many studies found a positive association, how many a negative, how many a null? With which specific marker? please try to avoid unspecific term such as “most of the studies” “in general” etc.

**R: We thank the reviewer for pointing out the need for clarification. Because of the great variability in the studies, it is a difficult task. We have now tried to summarize in the text the available literature identifying the number of biomarkers assessed in the different studies (considering the period assessed – pregnancy and/or postpartum) and the strength and direction of the association found between the inflammatory biomarkers and depression (self-reports vs. diagnoses) (please find in the text at page 13-14, line 321-330; page 14-15, line 344-360; page 16, line 394 and 398; page 17, line 417-423; page 18, line 443-447).**

**We have included the new suggested summary table we have constructed to summarize the results, as Supplementary file 3. If the Editor or the Reviewer think that we should not include it, we can omit it from the manuscript.**

9. Are there differences depending on: whether diagnosis of depression rather than self-reported depressive symptoms are used? Whether the samples are from high-risk or low-risk population, minority etc.? Biological fluids?

**R: Even after this new synthesis of the findings, we concluded that there is a great deal of methodological diversity and that there isn't a single identifiable factor (such as the study quality, biological fluid, timing, and format of evaluating the inflammatory response and depression, among other aspects analyzed and listed in the tables of results) that consistently enables us to explain the variations observed (page 21, line 501-502 and line 505-519; page 23, line 561-566; page 24-25, line 597-599). The Discussion section of this paper discusses this constraint and offers ideas for future research projects based on the considerations made here (please see page 26, line 624-634).**

10. Please include information about the quality assessment in the results section. This is only reported in the table. Are there differences in the association reported depending on the quality score of the paper?

**R: Thank you for this suggestion. A new paragraph was included in the results on the quality assessment process (page 12-13; line 297-309) including mean and standard deviation of the quality scores for each subgroup of studies regarding study design. Unfortunately, quality did not seem to influence presence or not of significant results. Even between studies with high quality, heterogenous results were found. This information was included in the Discussion section.**

11. Also, studies that analyzed the TRP/KYN pathway are included in the review but this has now been mentioned in the introduction. Please include it also in the introduction to allow readers from different fields to allow what these markers are and how they are related to the inflammatory function -this appear then in the discussion section but I would suggest to introduce it right at the beginning.

**R: We thank you for this recommendation. We have updated the Introduction with more information about the relationship between depression and inflammatory biomarkers, namely including the TRP/KYN pathway (please see page 4-5, line 97-107, in the revised manuscript).**

12. On page 15 it is stated This study also demonstrated that IL-6 and TNF -alpha levels at birth were predictors of symptomatology at 1 and 6 weeks postpartum? – which study?

**R: Thank you very much for having brought this typo to our attention. We have now identified the respective study in the manuscript (page 18, line 445-447), the text reading “Focusing on postpartum longitudinal prospective studies, a study (83) demonstrated that IL-6 and TNF -alpha levels at birth were predictors of symptomatology at 1 and 6 weeks postpartum.”**

Minor points:

1. Abstract: please add the sample size range of the studies included

**R: Thank you for this suggestion. We added the sample size range for cross-sectional and case-control studies to the Abstract, as well as for longitudinal studies (page 2, line 45-46).**
