# Supplementary material for: Inflammatory biomarkers and perinatal depression: A systematic review
Source: PLoS One. 2024 May 31;19(5):e0280612. doi: 10.1371/journal.pone.0280612 (PMC11142563; doi:10.1371/journal.pone.0280612)
Supplement: Attachment — Submitted filename: Response to Reviewers_March 19.docx [file pone.0280612.s006.docx]

Manuscript ID: PONE-D-23-00135

Title: Inflammatory biomarkers and perinatal depression: a systematic review

Journal: PLOS One

March 19, 2024

Dear Reviewers,

I would like to express our sincere gratitude for the thorough review on our manuscript titled "Inflammatory biomarkers and perinatal depression: a systematic review", particularly in light of the major revision request that prompted a reevaluation of our work. We would like to apologize for the late submission due to the Easter holiday and we deeply appreciate your time and effort dedicated to assessing our responses and the subsequent revisions.

We would like to extend our special thanks to Reviewer #1 for their insightful comments and positive feedback on the changes made by the authors in response to their initial review. Their acknowledgment of our efforts and encouragement regarding the significance of our contribution to the field are immensely gratifying.

Furthermore, we are grateful to Reviewer #3 for their diligent review and additional comments on the article. Their constructive feedback has been instrumental in further refining our manuscript and ensuring its quality and clarity.

We recognize the importance of the revision process in strengthening the scholarly impact of our work. A detailed description on how we address your comments is included below.

Yours sincerely,

Alkistis Skalkidou, MD, PhD

Professor of Obstetrics and Gynecology, Uppsala University, Sweden

Corresponding Author

**Comments and responses:**

**Journal requirements:**

Please review your reference list to ensure that it is complete and correct. If you have cited papers that have been retracted, please include the rationale for doing so in the manuscript text, or remove these references and replace them with relevant current references. Any changes to the reference list should be mentioned in the rebuttal letter that accompanies your revised manuscript. If you need to cite a retracted article, indicate the article's retracted status in the References list and also include a citation and full reference for the retraction notice.

**Reply: We thank you for this recommendation. The final draft of the manuscript was revised concerning the reference list to include all the cited papers.**

**Reviewer #1:**

All the suggested changes have been made. I believe it will be a paper that provides relevant information for the area.

**Reply: We sincerely appreciate the feedback of Reviewer #1. It's encouraging to hear that all the suggested changes have been implemented successfully. The recognition of our efforts reinforces our commitment to delivering valuable contributions to the field. We are dedicated to ensuring that our paper provides relevant and insightful information for the area. Thank you for your positive assessment.**

**Reviewer #3:**

This is a systematic review on the topic of inflammatory biomarkers and perinatal depression. There were 56 studies included where the primary aim of the studies were to assess associations between depression and inflammatory biomarkers. Their results support global hypotheses that PND and inflammatory biomarkers are associated, specifically IL-6, IL-8, CRP, TNF-alpha. They assessed and identified study heterogeneity on timing (and type?) of biological sampling, as well as timing and method for PND assessments. Strengths include their inclusion of publications in multiple languages. Weaknesses include that indiscriminate inclusion of biological fluids were included in the study, without discrimination on types of fluids/proteomics; excluded experimental studies, qualitative analyses, gray literature, case reports which can be helpful in providing hypothesis directions; data extraction did not report type of biological fluids.

**Reply: We thank the reviewer for highlighting the importance of discriminating between body fluids. We have now incorporated this information into the results, specifically in lines 297-299, which report the details already included in Table 1 and Table 2. Additionally, we have addressed this point in the discussion section, specifically in lines 576, 581-582.**

Major notes

• The methods are a systematic review, but per L190-194 they are asking specific questions about cross sectional associations and predictive questions about inflammation. Therefore a meta-analysis should possibly be performed and reported to answer these questions about magnitude of effects? Please, clarify or perform and report.

**Reply: Thank you for your thoughtful suggestion. We've carefully considered the possibility of performing a meta-analysis to address the specific questions raised regarding cross-sectional associations and predictive inquiries about inflammation. While we also acknowledge the potential benefits of such an analysis, and had considered it, we believe it may not be suitable for this study. As outlined in the revised objectives (L193-202), our focus is on conducting a systematic review to comprehensively synthesize the existing literature on the association between inflammation and perinatal depression. The decision not to pursue a meta-analysis is based on several factors, including the heterogeneous nature of the procedures followed in the studies we systematically reviewed, as discussed in the article (L613-624). Additionally, our primary aim is to provide a qualitative synthesis of the available evidence rather than quantifying effect sizes, which would not be realistic, as most studies have used different timepoints for measurement of exposure and outcome, and different analytical methods. We hope this clarifies our approach.**

• The methodology and reporting appears to be more consistent with a scoping review rather than a systematic review. Consider changing the title and revising throughout the document, that the study is a scoping review.

**Reply: Thank you for your insightful comment. We've carefully considered your suggestion and want to emphasize that our study adheres to the guidelines established by the PRISMA 2020 for conducting systematic reviews (http://www.prisma-statement.org/). We believe we've diligently followed these guidelines and have listed them in the PRISMA 2020 Checklist. Additionally, it's important to recognize that changing our approach to a scoping review would involve shifting the focus to a different methodology, which wasn't originally considered in the protocol registered and validated by Reviewer #1. However, we understand that our previous articulation of the objectives of our systematic review may have inadvertently caused confusion for readers. Therefore, we've revised the wording of the objectives (L193-202) to make them clearer and more aligned with the methodology we have actually followed. We hope this clarification addresses any concerns, and we remain open to further feedback or suggestions.**

• As a scoping review, more details on the types of studies performed and where existing knowledge gaps lie, would be informative.

**Reply: Thank you for your suggestion. We appreciate your interest in gaining more insight into the types of studies performed and identifying existing knowledge gaps. However, after careful consideration and review of our methodology, we believe that our study aligns with a systematic review rather than a scoping review. We believe that our study provides valuable insights into the topic at hand, as we do discuss existing knowledge gaps and future directions.**

• I disagree with the conclusion that there is a global need to harmonize methods. Currently, since there is so much unknown about the relationships and specificity between these inflammatory markers and PND, we need "discovery science" to flesh out potential mechanisms, timing, and other key things, before systematic reviews of an outcomes-based nature are appropriate. Please consider revising the Abstract and manuscript Conclusions. Notably, the scoping review that the authors conducted are important to identifying the differences in methods and current state of understanding on the topic, which can guide future research directions.

**Reply: We greatly appreciate the critical reflection provided by Reviewer #3. We partly agree with the reviewer on this matter and believe that both perspectives hold validity. It is crucial to strive for the harmonization of time points and methods to ensure comparability between studies and possibility for meta-analyses in the future. However, it is also essential to encourage the exploration of new methods and the search for new biomarkers for perinatal depression. We have now modified the Abstract (L54-64) and manuscript Conclusions (L643-649), to address the point raised by the Reviewer..**

Minor Notes

• The introduction is quite long and includes material that does not directly relate to the rationale for the study. It should be edited for brevity and focus.

**Reply: Thank you for your feedback. We acknowledge your observation regarding the length of the introduction and the inclusion of material that may not directly relate to the rationale for the study. We have taken your suggestion into consideration and made efforts to reduce the length of the introduction while maintaining focus on the rationale for the study and retaining the main essential ideas. We appreciate your input and believe that these revisions will enhance the clarity and focus of the Introduction.**

Specific Notes

• L80 "While epidemiological literature..." What does this mean? There is a lot of literature on perinatal mental health that goes beyond describing what is happening or rates/incidences of certain phenomena. Please be specific or cut this clause out.

**Reply: Thank you for your comment. We agree that the clause "While epidemiological literature..." may not be necessary and could potentially confuse the idea we are trying to convey. We appreciate your input and have accordingly removed this clause.**

• L182-189 This is a run-on sentence. Please edit.

**Reply: Thank you for your feedback. We appreciate your attention to detail. The run-on sentence you mentioned has been addressed, and the revised formulation can now be found at L193-202. If you have any further suggestions or concerns, please feel free to let us know.**
